# Time trends in colorectal cancer incidence across the BRICS: an age-period-cohort analysis for the GBD 2021

**DOI:** 10.3389/fonc.2025.1633242

**Published:** 2025-11-04

**Authors:** Chengcheng Zhang, Linzhi Chen, Chun Zhang, Yuqi Xiu, Hongling Zhang, Wenjuan Ying, Hui Liu

**Affiliations:** ^1^ Institute of Nursing Research, The First Affiliated Hospital of Shantou University Medical College, Shantou, Guangdong, China; ^2^ Department of Nursing, Shantou University Medical College, Shantou, Guangdong, China; ^3^ Orthopedics Department, Cancer Hospital of Shantou University Medical College, Shantou, Guangdong, China; ^4^ Inpatient Area of Interventional Ultrasound, Cancer Hospital of Shantou University Medical College, Shantou, Guangdong, China

**Keywords:** colorectal cancer, incidence, age-period-cohort mode, BRICS, trend

## Abstract

**Background:**

Colorectal cancer (CRC) is a leading global health burden, contributing significantly to disability-adjusted life years and economic burden. The BRICS nations—spanning diverse and rapidly evolving socio-economic contexts—are undergoing critical epidemiological transitions. Understanding CRC trends in these countries is essential to inform targeted control strategies.

**Methods:**

Data from the Global Burden of Disease (GBD) 2021 database were used to assess trends in CRC incidence across BRICS countries from 1990 to 2021. An age-period-cohort (APC) model with the intrinsic estimator (IE) algorithm was employed to disentangle the independent effects of age, period, and cohort on incidence rates. Data were stratified into 5-year age groups, and 95% uncertainty intervals (UIs) were calculated to reflect variability and estimation precision.

**Results:**

From 1990 to 2021, the global CRC cases increased by 139.38%, with the age-standardized incidence rate (ASIR) rising by 6.52%. Among BRICS nations, Saudi Arabia had the largest increase in cases (111.02%), while United Arab Emirates showed a decline (-23.04%). Globally, most age groups exhibited positive local drift values, indicating rising incidence rates, except for individuals under 20 years. This pattern was also observed in India and South Africa, whereas Ethiopia showed a distinct trend. Brazil, China, Egypt, Iran, and Saudi Arabia experienced consistent increases across nearly all age groups. The age effect revealed a low CRC risk before age 35–39, with risk rising steadily and peaking at age 90–94, a pattern consistent across all countries. Period effects were relatively stable globally, with increasing trends in all BRICS nations except Ethiopia. Cohort effects generally increased over time, stabilizing in recent birth cohorts, with a steeper rise among males. However, India and Ethiopia showed declining cohort risks.

**Conclusion:**

This study highlights a substantial global increase in CRC incidence, with notable variations across BRICS nations over the past three decades. The observed age, period, and cohort effects underscore the need for age-specific and gender-sensitive health policies. Ongoing surveillance, research, and targeted public health interventions are critical to mitigating the rising CRC burden and improving health outcomes in these rapidly evolving regions.

## Introduction

Colorectal cancer (CRC), originating in the colon or rectum, remains one of the leading causes of cancer-related morbidity and mortality worldwide (1, 2). While the incidence is highest in developed nations, a rising trend has been observed across many low- and middle-income countries, underscoring its growing global health impact (3). Most CRC cases arise sporadically, often developing from dysplastic adenomatous polyps (4). During disease progression, metastasis to the liver and lungs occurs in 40–50% of patients, and approximately one-quarter of individuals present with liver metastases at diagnosis, indicating late-stage detection in many cases (5, 6). Established risk factors include inflammatory bowel disease, family history of CRC, elevated body mass index, smoking, sedentary lifestyles, and specific dietary patterns (7). Despite advances in screening and treatment, the prognosis remains poor for patients diagnosed at advanced stages (8). A comprehensive understanding of CRC epidemiology is therefore critical for informing prevention strategies, optimizing healthcare resource allocation, and improving outcomes.

Emerging economies are increasingly central to the global cancer burden due to rapid demographic and socioeconomic transitions, shifts in lifestyle, and evolving healthcare systems (9). Traditionally, the BRICS nations comprised Brazil, Russian Federation, India, China, and South Africa (10). However, as of January 1, 2024, the group has expanded to include Saudi Arabia, Egypt, the United Arab Emirates, Iran, and Ethiopia, forming a broader bloc often referred to as ‘BRICS-plus’ (11, 12). This ten-nation bloc represents a substantial portion of the global population and disease burden. Although these countries differ in geography and culture, they share common challenges, such as urbanization, aging populations, healthcare infrastructure disparities, and increasing exposure to modifiable CRC risk factors (13). However, systematic and comparative assessments of CRC incidence across this expanded BRICS group remain scarce, limiting efforts to identify disparities and guide policy development in these settings.

The Global Burden of Disease (GBD) 2021 study offers a robust and standardized framework for evaluating CRC burden across time and geography, incorporating data on incidence, mortality, and risk factors from a wide range of global sources (14). Leveraging such data, the age–period–cohort (APC) model enables a nuanced examination of temporal trends by disentangling the effects of biological aging, time-specific factors (e.g., screening practices or treatment advances), and generational shifts in risk exposure (15). Although previous analyses using GBD data have offered valuable insights, they have often lacked the resolution required for national-level decision-making and have rarely explored within-country heterogeneity—particularly among BRICS nations (16, 17). Addressing this gap is essential for designing context-specific interventions that can effectively target country-level trends.

In this study, we utilize the most recent GBD 2021 dataset to perform a comprehensive APC analysis of CRC incidence trends in BRICS countries from 1990 to 2021. By examining variations across age groups, calendar periods, and birth cohorts, we aim to characterize the evolving epidemiology of CRC at the national level. Our findings provide critical insights into demographic and temporal drivers of CRC incidence, which can support the development of targeted public health strategies, promote equitable cancer control, and contribute to reducing the burden of CRC in these rapidly transforming regions.

## Method

### Data sources

This study used data from the GBD 2021 public dataset, accessible via the Global Health Data Exchange (GHDx) GBD Results Tool (https://ghdx.healthdata.org/gbd-2021). The GBD 2021 provides comprehensive estimates for 371 diseases and injuries across 204 countries and territories worldwide (18). The most recent iteration includes significant updates: integration of 19,189 additional data sources for disability-adjusted life years, inclusion of 12 newly recognized health conditions, and multiple methodological refinements. Furthermore, it incorporates the impact of the COVID-19 pandemic on the global disease burden (19).

We extracted data on the number of incident numbers, all-age incidence rates, and age-standardized incidence rates (ASIR) for CRC at both global and BRICS country levels, stratified by age groups ranging from <5 years to ≥95 years, for the period 1990 to 2021. In this study, “Global” denotes estimates for the entire world, encompassing 204 countries and territories as provided in GBD 2021, not limited to BRICS. All estimates were accompanied by 95% uncertainty interval (UI), calculated from 1,000 draws from the posterior distribution, with the 2.5th and 97.5th percentiles defining the bounds (20).

Detailed descriptions of GBD 2021 methodology and modeling strategies are available in previously published sources (14, 18). The data used in this study were de-identified and publicly available, therefore, the requirement for informed consent was waived, as approved by the Institutional Review Board of the University of Washington. According to the list of International Classification of Diseases (ICD) codes mapped to non-fatal causes and injuries in GBD 2021, colon and rectum cancer was defined using ICD-10 codes C18–C19.0, C20, and C21–C21.8 (21).

### Statistical analysis

#### Age-period-cohort modelling analysis

To examine temporal trends in CRC incidence, we applied an APC analytical framework, modeling CRC incidence as the dependent variable under the assumption of a Poisson distribution. Age, period, and cohort were included as independent variables. The APC model is designed to disentangle the separate effects of aging (age effect), time-related factors affecting all age groups (period effect), and generational exposures linked to birth year (cohort effect) (22).

Specifically, the age effect captures variations in CRC risk attributable to biological and behavioral changes associated with aging. The period effect reflects contemporaneous influences—such as the introduction of screening programs or advances in medical care—that impact all age groups simultaneously. The cohort effect accounts for differences in risk arising from exposures or risk factors specific to particular birth cohorts (e.g., changes in diet, lifestyle, or early-life environment) (23).

To address the inherent identification problem arising from the exact linear dependency among age, period, and cohort (i.e., cohort = period − age), we applied the intrinsic estimator method. This approach is widely recognized as a statistically robust and unbiased solution to the non-identifiability issue inherent in APC models. Its validity and reliability have been demonstrated in multiple prior studies (24). The main output indicators of the APC model included net drift, local drift, the longitudinal age curve, and relative risks by period and cohort (25). Net drift represents the overall annual percent change in CRC incidence across the population. Local drift measures age-specific trends. A positive local drift indicates rising incidence in specific age groups, while a negative local drift reflects a decline in those age groups. The longitudinal age curve presents age-specific incidence rates for a reference cohort, adjusted for period effects. Period RR and cohort RR quantify the relative risk across time periods and birth cohorts, respectively, adjusting for age and the other temporal variable.

#### Data arrangement

To control model complexity while maintaining smooth temporal trends, age-specific CRC incidence rates were grouped into 5-year age intervals (<5, 5–9, 10–14,…, ≥95 years). In accordance with standard APC modeling practices, both age and period were structured using uniform 5-year intervals, consistent with the GBD dataset. This approach balances trend capture with model simplicity, ensuring stability, cross-temporal and cross-country comparability, and mitigating nonidentifiability from unequal intervals (26). However, rather than using 5-year averages to represent calendar periods, we integrated data from the GBD study by extracting incidence and population estimates from the mid-year of six specific time points: 1992, 1997, 2002, 2007, 2012, and 2017. Birth cohorts were derived by subtracting age from period (cohort = period − age), and ranged from individuals born between 1911 and 1919 (median birth year 1915) to those born between 1991 and 1999 (median birth year 1995). The 1952–1962 birth cohort was selected as the reference group because it is centrally located within the cohort range, ensuring statistical balance. This cohort also reflects a period of relative stability in exposures and healthcare access, serving as a robust reference to enhance model stability and interpretability.

Parameter estimation for the APC analysis was conducted using the web-based APC tool developed by the National Institutes of Health (NIH) (https://analysistools.cancer.gov/apc/) (26). Visualization of model outputs was performed using the ggplot2 package in R (version 4.2.3) (27, 28). The input data included age-specific incidence counts and population denominators formatted as a rate matrix with paired columns. Model outputs comprised estimators of cross-sectional and longitudinal age-specific incidence rates, period and cohort rate ratios adjusted for net drift (the overall annual percentage change), and local drift values reflecting age-specific annual percentage changes. Statistical significance of the model parameters and derived functions was assessed using the Wald *χ* ^2^ test, with all tests being two-sided. An alpha level of 0.05 was used to determine statistical significance.

## Results

**Table 1** presents the population, total number of incidence, all age incidence rate, ASIR, and net drift of CRC incidence. Globally, the number of incident CRC cases increased from 917,000 (95% UI: 866,000–952,000) in 1990 to 2,194,000 (95% UI: 2,001,000–2,359,000) in 2021, representing a 139.38% increase. The global ASIR also increased from 24.04 (95% UI: 22.54–25.01) in 1990 to 25.61 (95% UI: 23.32–27.52) per 100,000 population in 2021, reflecting a relative increase of 6.52%. Based on the APC model, the estimated global net drift in CRC incidence was 0.15% per year (95% CI: 0.12–0.19) from 1990 to 2021 (**Table 1**).

**Table 1:** Trends in colorectal cancer incidence across global and BRICS, 1990–2021.

Location	Population		Incidences			All-age incidence rate		Age-standardized incidence rate		APC model estimates
	Number, n × 1,000,000	Percentage of global, %	Number, n×1,000	Percentage of global, %	Percent change of number 1990–2021, %	Rate per 100,000	Percent change of rate 1990–2021, %	Rate per 100,000	Percent change of rate 1990– 2021, %	Net drift (% per year, 95%CI)
Global										
1990	5334(5231,5445)	100.0	917(866,952)	100.0	139.38	17.19(16.24,17.85)	61.79	24.04(22.54,25.01)	6.52	0.15(0.12,0.19)
2021	7891(7667,8131)	100.0	2194(2001,2359)	100.0		27.80(25.36,29.90)		25.61(23.32,27.52)		
Brazil										
1990	149(138,159)	2.78	10(9,10)	1.06	348.04	6.52(6.20,6.85)	201.96	11.10(10.42,11.68)	55.21	1.39(1.27,1.51)
2021	220(188,251)	2.79	43(40,46)	1.98		19.69(18.18,21.00)		17.23(15.86,18.39)		
China										
1990	1176(1097,1264)	22.06	158(135,183)	17.28	315.3	13.46(11.51,15.52)	243.69	19.04(16.46,21.81)	65.13	1.74(1.63,1.85)
2021	1423(1319,1530)	18.03	658(532,798)	30		46.27(37.39,56.09)		31.44(25.53,37.97)		
Egypt										
1990	55(50,61)	1.04	2(2,2)	0.19	355.40	3.22(2.87,3.61)	138.56	6.32(5.59,7.20)	98.87	2.78(2.52,3.04)
2021	106(96,116)	1.34	8(7,10)	0.37		7.69(6.27,9.43)		12.57(10.41,15.33)		
Ethiopia										
1990	51(46,56)	0.95	4(2,5)	0.44	67.52	7.91(4.41,9.77)	-22.24	21.32(12.46,26.19)	-23.04	-1.16(-1.38,-0.93)
2021	109(92,125)	1.38	7(6,8)	0.31		6.15(5.12,7.38)		16.41(13.70,19.54)		
India										
1990	853(789,915)	15.99	23(19,25)	2.46	207.95	2.64(2.25,2.99)	85.72	4.61(3.89,5.25)	23.60	0.56(0.39,0.72)
2021	1414(1240,1602)	17.92	69(62,79)	3.16		4.91(4.37,5.55)		5.69(5.05,6.45)		
Iran (Islamic Republic of)										
1990	57(52,62)	1.07	2(2,3)	0.25	349.54	4.06(3.38,4.70)	200.73	8.93(7.51,10.30)	47.74	1.67(1.44,1.90)
2021	85(77,94)	1.08	10(9,12)	0.48		12.21(10.57,13.68)		13.19(11.44,14.76)		
Russian Federation										
1990	151(139,163)	2.83	44(42,45)	4.75	88.54	28.81(27.88,29.59)	96.52	23.98(23.17,24.60)	42.56	1.07(0.92,1.21)
2021	145(125,164)	1.84	82(75,89)	3.74		56.62(51.85,61.31)		34.18(31.30,37.02)		
Saudi Arabia										
1990	16(14,17)	0.3	0(0,1)	0.05	695.28	2.74(1.96,3.62)	234.45	7.05(5.15,9.25)	111.02	2.75(2.43,3.06)
2021	38(33,43)	0.48	3(3,4)	0.16		9.18(7.03,11.48)		14.88(12.12,18.16)		
South Africa										
1990	37(33,41)	0.69	2(2,2)	0.21	219.86	5.18(4.53,6.52)	108.23	9.29(8.04,11.89)	44.93	1.32(1.12,1.52)
2021	57(50,64)	0.72	6(5,7)	0.28		10.79(9.65,12.12)		13.46(12.07,14.96)		
United Arab Emirates										
1990	2(2,2)	0.04	0(0,0)	0.01	510.39	5.57(3.67,7.66)	18.57	21.16(14.40,29.03)	-11.21	1.19(0.69,1.70)
2021	10(8,11)	0.12	1(0,1)	0.03		6.60(4.74,9.77)		18.79(14.27,26.88)		

Values in parentheses denote 95% uncertainty intervals (UIs) for GBD-derived estimates. For APC-derived net drift, values in parentheses denote 95% confidence intervals (CIs) obtained from Wald tests. Net drift of incidence rate represents the overall annual percentage change in incidence estimated by the APC model. APC, age-period-cohort.

From 1990 to 2021, considerable variation in ASIR was observed across BRICS countries. The largest increases were seen in Saudi Arabia, Egypt, and China. In Saudi Arabia, the ASIR rose from 7.05 (95% UI: 5.15–9.25) to 14.88 (95% UI: 12.12–18.16) per 100 000 population, an increase of 111.0%. Egypt experienced a similar rise, with ASIR increasing from 6.32 (95% UI: 5.59–7.20) in 1990 to 12.57 (95% UI: 10.41–15.33) per 100,000 population in 2021, a 98.9% increase. In China, the ASIR rose from 19.04 (95% UI: 16.46–21.81) to 31.44 (95% UI: 25.53–37.97) per 100,000 population, a 65.13% increase. In contrast, Ethiopia and the United Arab Emirates showed declining trends. In Ethiopia, the ASIR decreased from 21.32 (95% UI: 12.46–26.19) in 1990 to 16.41 (95% UI: 13.70–19.54) per 100,000 population in 2021, a 23.04% reduction. In the United Arab Emirates, the ASIR dropped from 21.16 (95% UI: 14.40–29.03) to 18.79 (95% UI: 14.27–26.88) per 100,000 population, a decrease of 11.2%. According to APC model estimates, the annual net drift in CRC incidence ranged from -1.16% (95% CI: -1.38, -0.93) in the Ethiopia to 2.78 (95% CI: 2.52, 3.04) in Egypt among BRICS countries (**Table 1**).

### Time trends in colorectal cancer incidence across different age groups

**Figure 1:**
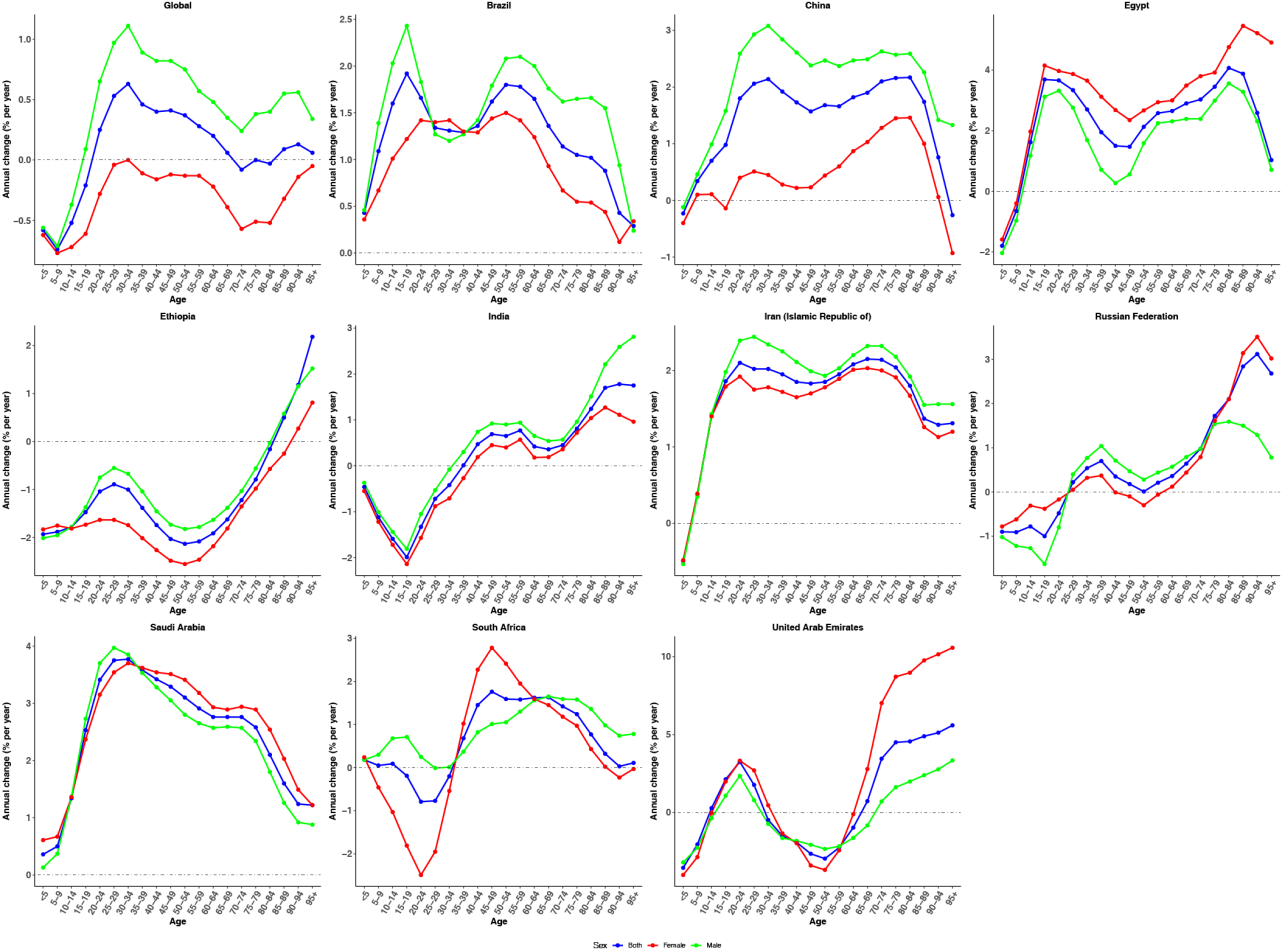
The local drifts of CRC incidence rate in global and BRICS, 1990-2021. Local drifs of CRC incidence rate (estimates from age-period-cohort models) for age groups (0–4, 5–9, 10–14, …, 95+ years), 1990-2021. The dots indicate the annual percentage change of incidence rate (% per year).

**Figure 1** presents the estimated annual percentage change in ASIR of CRC by age group from 1990 to 2021. Globally, most age groups exhibited positive local drift values, indicating an overall increase in CRC incidence. An exception was observed among the pediatric and adolescent populations (<5, 5–9, 10–14, and 15–19 years), where negative local drift values reflected a declining trend over time. Males consistently demonstrated higher estimated annual percentage change values across all age groups compared to females, suggesting a more pronounced increase in CRC incidence among men. Country-specific trends revealed distinct age-related patterns. In India and South Africa, increases in CRC incidence were primarily concentrated among individuals aged ≥35 years, while younger age groups showed declining trends. In contrast, Ethiopia exhibited a nearly universal decrease across all age groups, with negative local drift values except for the oldest age group (≥85 years). Conversely, Brazil, China, Egypt, Iran, and Saudi Arabia demonstrated consistent upward trends, with positive local drift values observed across nearly all age categories. These findings highlight a widespread and increasing burden of CRC across the life course in these nations, particularly in middle-aged and older adults.

**Figure 2:**
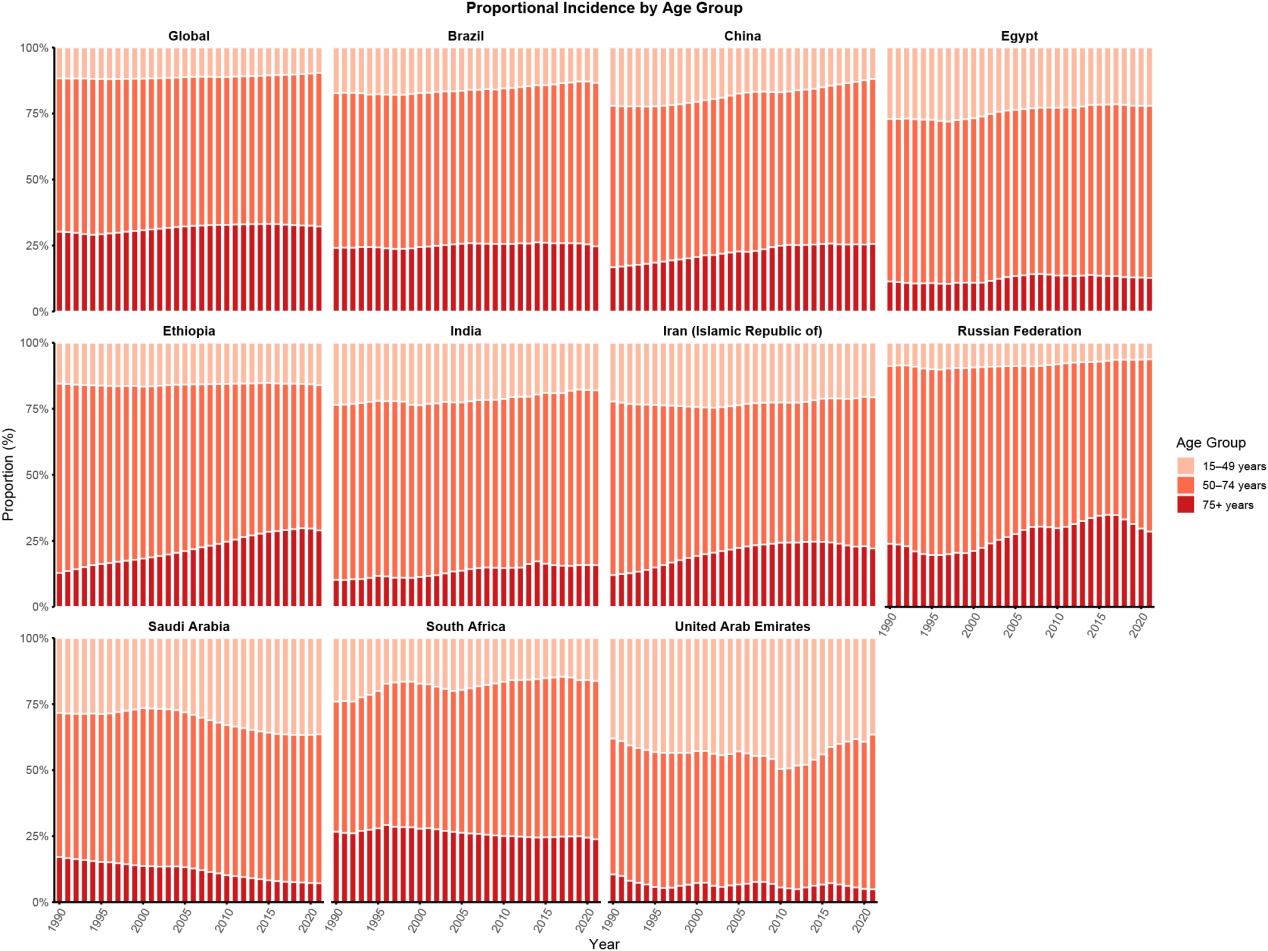
Age distribution of incidence from CRC in global and BRICS, 1990-2021. Age distribution of incidence is represented as temporal change in the relative proportion incidence across age groups (15–49, 50–74, and 75+ years) during 1990-2021. The 0–4 and 5–14 age groups showed 0% incidence globally and in each BRICS country and are therefore not displayed.

**Figure 2** illustrates temporal changes in the age distribution of CRC incidence between 1990 and 2021. At the global level, the age-specific proportion of CRC cases remained relatively stable, with individuals aged 50–74 years consistently accounting for the highest burden. This pattern was similarly observed in Brazil, Egypt, and India. However, several countries showed notable deviations. In Ethiopia, a discernible shift in CRC incidence was observed, with the distribution transitioning from the middle-aged population (50–74 years) toward the older age group (≥75 years). A similar redistribution was observed in China, characterized by a shift from younger individuals (15–49 years) to the elderly population (≥75 years), indicating an increasing proportion of CRC cases among older adults over time. In contrast, Saudi Arabia and the United Arab Emirates exhibited a similar but distinct trend, with the CRC burden shifting from older adults (≥ 75 years) toward the 50–74 and 15–49 year age groups.

### Age, period and cohort effects on colorectal cancer incidence

**Figure 3:**
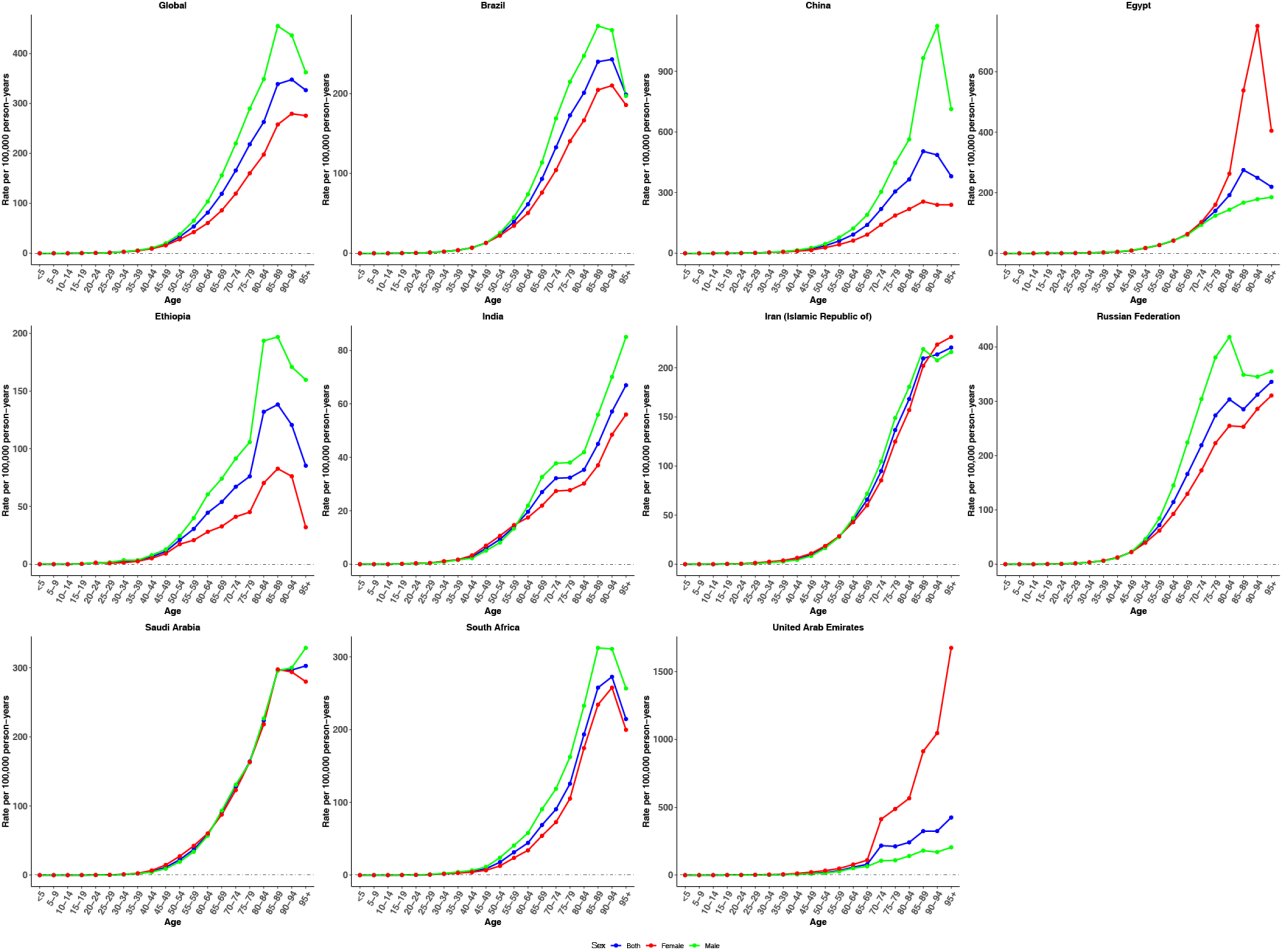
Age effects on CRC incidence in global and BRICS. Longitudinal age curves of incidence rate (per 100,000 person-years), adjusted for period deviations.

**Figure 4:**
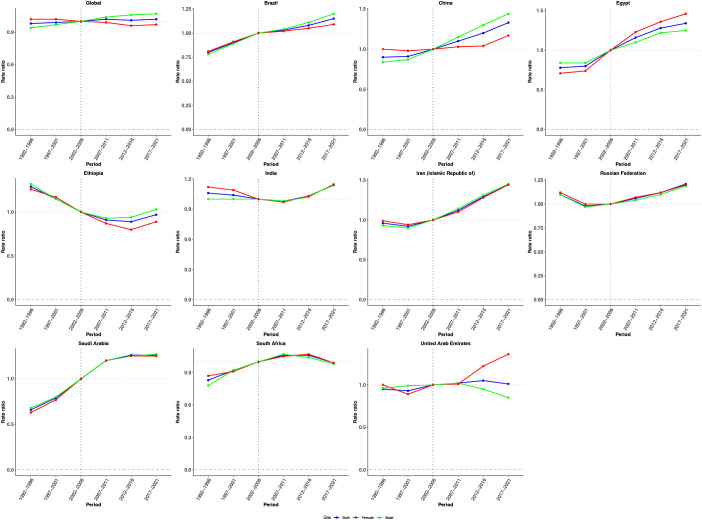
Period effects on CRC incidence in global and BRICS. Relative risk (incidence rate ratio) computed as the ratio of age-specific rates between 1992–1996 and 2017–2021, with 2002–2006 as the referent period.

**Figure 5:**
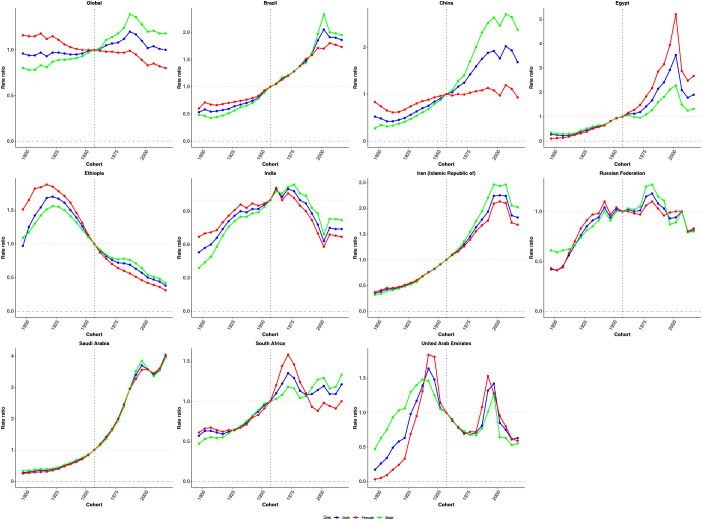
Cohort effects on CRC incidence in global and BRICS. Relative risk computed as the ratio of age-specific rates between the 1897 and 2017 cohorts, with 1957 as the referent cohort. Dots and shaded areas represent incidence rates or rate ratios and their 95% CIs.

**Figures 3**–**5** show the APC effects estimates derived from the APC model by global and BRICS countries. Globally, incidence risk remained relatively low before the 35–39 age group but rose steadily thereafter, peaking in the 90–94 age group. This trend underscores the heightened vulnerability of older adults to CRC. Overall, a similar age effect pattern is observed across all nations, with risk increasing as age increases (**Figure 3**). These findings suggest a shared pattern of age-related risk accumulation, despite heterogeneity in demographic and environmental exposures. Notably, with the exception of Egypt and the United Arab Emirates, males consistently exhibited higher age-specific risk compared to females.

**Figure 4** illustrates the estimated period effects on CRC incidence. Globally, the period effects remained relatively stable over the past three decades, suggesting limited variation in CRC incidence risk over time. A comparable trend was observed in the United Arab Emirates. With the exception of Ethiopia, all other countries demonstrated an increasing trend in period effects relative to the reference period, indicating a gradual rise in CRC incidence risk during the observation window. In contrast, Ethiopia showed a distinct decline. Regarding sex-specific patterns, males consistently showed higher period effect ratios globally. This disparity was especially evident in Brazil and China, where the period effects for males significantly exceeded those for females from 2007–2011 to 2017–2021, compared with the reference period (2002–2006), indicating a greater CRC incidence burden among men during the study period.

Cohort effects exhibited an overall increasing trend globally, followed by stabilization in more recent birth cohorts, with a slightly steeper rise observed among males (**Figure 5**). Brazil, China, Egypt, the Islamic Republic of Iran, Saudi Arabia, and South Africa showed sustained upward trends across successive birth cohorts, particularly after the reference cohort (1952–1962). In contrast, Ethiopia and India demonstrated a declining pattern in cohort risk over time. The Russian Federation and the United Arab Emirates presented a fluctuating trajectory, with initial increases followed by subsequent declines in cohort effects.

## Discussion

This study applies the APC model to systematically analyze temporal trends in CRC incidence at both global and BRICS country levels. Compared with prior analyses based on GBD data (16, 17, 29), our primary contribution lies in disentangling the distinct contributions of age, period, and cohort effects to observed incidence trends. Additionally, we estimated local drift values across age groups and tracked age-specific incidence redistributions, offering a more nuanced understanding of shifting CRC dynamics from 1990 to 2021. These analytical innovations provide actionable insights for policymakers and public health professionals, particularly in designing prevention strategies tailored to specific age groups and birth cohorts.

Between 1990 and 2021, global CRC incidence rose by 139.38%, accompanied by a 6.52% increase in the ASIR. This upward trend is primarily attributed to population aging, lifestyle-related risk factors (including poor diet, sedentary behavior, and obesity), and improved early detection (30, 31). Despite advances in diagnosis and treatment, significant disparities persist in prevention, early detection, and timely access to treatment, particularly in low- and middle-income countries, thereby contributing to the continued rise in global CRC incidence. The global net drift of 0.15% per year, along with predominantly positive local drift values across age groups, suggests that the increase in incidence reflects a true elevation in generational risk, rather than demographic changes alone. The relative stability of period effects at the global level indicates limited progress in population-wide screening and diagnostic interventions over the past three decades. Cohort effects, particularly among individuals born after 1970, showed an overall increase before leveling off in more recent birth cohorts. This pattern is plausibly linked to greater exposure to modifiable lifestyle factors—such as Westernized dietary patterns, reduced physical activity, and rising obesity (32). The subsequent plateau may reflect a stabilization of these exposures alongside earlier detection as public awareness improved and screening programs expanded (33, 34). Globally, males consistently exhibit higher CRC incidence than females, likely reflecting sex-specific differences in behavior, metabolism, and biology—on average, men have greater lifetime exposure to tobacco and alcohol, more central adiposity with adverse metabolic profiles, lower screening participation, and potentially weaker hormonal protection (35, 36).

Our analysis reveals substantial regional heterogeneity in CRC incidence. Among BRICS countries, Saudi Arabia, Egypt, and China experienced the most pronounced increases in ASIR, with Saudi Arabia reporting a striking 111.02% rise. These increases are largely driven by rapid urbanization, shifts in lifestyle (e.g., increased consumption of high-fat, low-fiber diets), and aging populations (37). In Saudi Arabia, CRC has become the most common malignancy among men and the third most common among women, with over 66% of cases diagnosed at advanced stages (38). Contributing factors include widespread physical inactivity, high obesity prevalence, and limited public awareness of screening programs (39). Egypt and China also experienced marked ASIR increases, underscoring the role of lifestyle changes and demographic transitions in escalating CRC burden. In contrast, Ethiopia demonstrated a 23.04% decline in ASIR and a negative net drift–possibly reflecting a youthful population structure, incomplete cancer registration, and low screening coverage (40). The United Arab Emirates similarly exhibited a modest decline in ASIR, potentially due to demographic shifts, including a large influx of younger migrant workers, as well as underreporting and diagnostic delays linked to underdeveloped cancer surveillance systems (41). However, these observed declines may not necessarily reflect a true reduction in disease burden but rather underscore the need for enhanced cancer registry systems and improved surveillance accuracy.

APC trajectories in Brazil, China, Egypt, Iran, and Saudi Arabia consistently revealed rising ASIR, positive net drift, and pronounced cohort effects, particularly among individuals born after 1970. These patterns reflect the convergence of epidemiological transitions with regional risk exposures such as dietary Westernization, increased obesity prevalence, and insufficient early screening (42). In China and Egypt, the CRC burden has increasingly shifted toward older adults due to both population aging and expanded healthcare access. These findings underscore the need to strengthen organized screening for adults ≥ 60 years—especially men, who have lower uptake–and to expand colonoscopy/fecal immunochemical test (FIT) coverage through insured primary care (43). In contrast, Saudi Arabia and the United Arab Emirates showed a trend toward earlier-onset CRC, with increasing incidence among individuals aged 15–49 years. This concerning shift highlights the need to revisit current screening guidelines, which often exclude younger age groups despite rising risk. In these settings, earlier screening may be justified where local risk, capacity, and cost-effectiveness permit (44, 45). Brazil also faces a growing CRC burden, particularly among younger adults, likely due to urbanization-related lifestyle changes and delayed implementation of national screening programs (46). During program expansion, opportunistic coverage of younger mid-adult ages may serve as a pragmatic interim approach, together with interventions to reduce obesity and sedentary time in young men (47, 48). Across these countries, the APC model consistently demonstrates steep age effects and amplified cohort effects—suggesting that recent generations face higher risks driven by cumulative exposure to carcinogenic behaviors and environments (49). These findings emphasize the need for generation-specific interventions and the integration of CRC prevention into broader non-communicable disease strategies.

India and Ethiopia exhibited declining cohort effects and negative net drift. In India, early public health interventions—such as the National Cancer Control Program, which emphasizes education and primary prevention—may have contributed to this trend. Notably, pilot projects implemented under the program that integrated community education, primary-care FIT, and clear referral/navigation pathways have reported higher screening completion (50). However, the absence of a nationwide, population-based CRC screening program limits interpretability, as undetected or unreported cases may obscure the true disease burden (51). Ethiopia’s declining CRC incidence likely reflects a combination of demographic and healthcare system factors. The nation’ s predominantly young population lowers overall CRC risk, while limited healthcare infrastructure contributes to underreporting and underdiagnosis. Restricted access to medical services, substantial urban-rural disparities, and limited diagnostic capacity further reinforce this pattern. Moreover, the absence of population-based CRC screening and incomplete cancer registration may obscure the true disease burden, a challenge commonly observed in low-resource settings (52, 53). Beyond these health-system considerations, the observed declines in both countries may also be consistent with cohort-level shifts in exposures—toward healthier dietary patterns, more favorable physical-activity/adiposity trajectories, reduced tobacco/alcohol uptake, and improved early-life environments (54).

South Africa and the Russian Federation displayed more complex APC patterns. In South Africa, the observed increase in cohort effects and positive drift among older adults may reflect rising life expectancy and improved registry coverage through the South African National Cancer Registry (55). The Russian Federation showed a fluctuating cohort pattern—initially increasing then decreasing—possibly influenced by historical clinical screening policies and recent changes in healthcare access. Nonetheless, the persistent elevation in CRC risk among younger males in Russia suggests emerging exposures such as alcohol, tobacco, and processed food consumption warrant further investigation (56). The United Arab Emirates demonstrated a unique APC trajectory. While the age effect followed expected patterns, cohort effects indicated a shift in risk toward individuals born after 1980. This trend may be linked to demographic changes and increasing adoption of Westernized lifestyles in a highly mobile population (57). The relatively flat period effects suggest that recent healthcare reforms have yet to meaningfully impact CRC incidence trends.

Several limitations should be acknowledged. First, national-level data may obscure subnational disparities, particularly in countries with heterogeneous access to healthcare and varying socioeconomic conditions. In addition, data quality varies across BRICS—for example, incomplete cancer registries in Ethiopia may underestimate true incidence, while migration patterns such as the influx of young migrant workers in the UAE could distort age-specific trends and cohort analyses (41). Second, although GBD estimates are standardized, variability in data quality and diagnostic practices across countries may introduce bias. Third, the use of five-year intervals in the APC model may limit detection of subtle temporal trends, especially for early-onset CRC. Moreover, APC analyses are ecological and not designed for causal inference. They cannot separate the effects of diet, screening uptake, and healthcare expansion. Follow-up studies using individual-level or longitudinal data are needed to clarify these relationships. Finally, recent public health shifts and interventions may not yet be fully reflected in our study period. Future studies should incorporate longitudinal cohort datasets, subnational analyses, and country-specific APC models to further elucidate evolving CRC risk patterns in the BRICS nations and beyond.

## Conclusion

In general, this study provides a comprehensive analysis of CRC incidence trends from 1990 to 2021 using an age–period–cohort framework across global and BRICS contexts. By delineating the independent contributions of age, period, and cohort effects, we reveal that the increasing CRC burden is not solely attributable to demographic shifts but reflects rising generational risk, particularly among individuals born after 1970. These findings highlight significant epidemiological transitions in rapidly developing economies, including a shift toward earlier-onset CRC in some settings. The persistence of positive local and net drifts, especially in countries such as China, Saudi Arabia, and Egypt, underscores the urgent need for context-specific, generation-targeted prevention strategies, as well as a re-examination of existing screening guidelines to encompass younger populations. Our results also underscore the importance of strengthening cancer surveillance systems and expanding equitable access to early detection and treatment. Future efforts should prioritize longitudinal, subnational, and policy-integrated analyses to further inform tailored interventions aimed at reversing the global rise in CRC incidence.

## Data Availability

The original contributions presented in the study are included in the article/supplementary material. Further inquiries can be directed to the corresponding authors.
